# Atomic-Scale Insights into Nickel Exsolution on LaNiO_3_ Catalysts via *In Situ* Electron Microscopy

**DOI:** 10.1021/acs.jpcc.1c09257

**Published:** 2021-12-30

**Authors:** Pengfei Cao, Pengyi Tang, Maged F. Bekheet, Hongchu Du, Luyan Yang, Leander Haug, Albert Gili, Benjamin Bischoff, Aleksander Gurlo, Martin Kunz, Rafal E. Dunin-Borkowski, Simon Penner, Marc Heggen

**Affiliations:** † School of Chemical Engineering and Technology, Xi’an Jiaotong University, Xi’an 710049, China; ‡ Ernst Ruska-Centre for Microscopy and Spectroscopy with Electrons, Forschungszentrum Jülich GmbH, Leo-Brandt-Strasse 1, D-52428 Jülich, Germany; § Department of Physical Chemistry, University of Innsbruck, Innrain 52c, A-6020 Innsbruck, Austria; ∥ Chair of Advanced Ceramic Materials, Institut für Werkstoffwissenschaften und -technologien, Technical University Berlin, Hardenbergstrasse 40, D-10623 Berlin, Germany; ⊥ Advanced Light Source, Lawrence Berkeley National Laboratory, Berkeley, California 94720, United States; # State Key Laboratory of Information Functional Materials, 2020 X-Lab, ShangHai Institute of Microsystem and Information Technology, Chinese Academy of Sciences, Shanghai, 200050, P. R. China

## Abstract

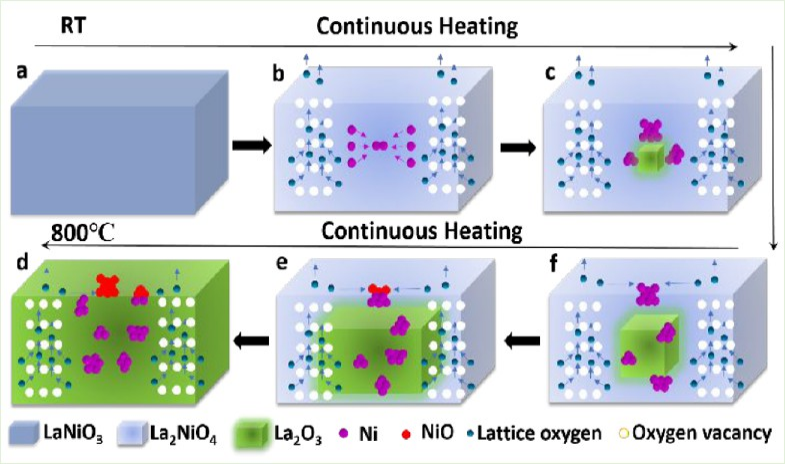

Using a combination of *insitu* bulk and surface characterization techniques, we provide atomic-scale insight into the complex surface and bulk dynamics of a LaNiO_3_ perovskite material during heating *in vacuo*. Driven by the outstanding activity LaNiO_3_ in the methane dry reforming reaction (DRM), attributable to the decomposition of LaNiO_3_ during DRM operation into a Ni//La_2_O_3_ composite, we reveal the Ni exsolution dynamics both on a local and global scale by *insitu* electron microscopy, *insitu* X-ray diffraction and *insitu* X-ray photoelectron spectroscopy. To reduce the complexity and disentangle thermal from self-activation and reaction-induced effects, we embarked on a heating experiment in vacuo under comparable experimental conditions in all methods. Associated with the Ni exsolution, the remaining perovskite grains suffer a drastic shrinkage of the grain volume and compression of the structure. Ni particles mainly evolve at grain boundaries and stacking faults. Sophisticated structure analysis of the elemental composition by electron-energy loss mapping allows us to disentangle the distribution of the different structures resulting from LaNiO_3_ decomposition on a local scale. Important for explaining the DRM activity, our results indicate that most of the Ni moieties are oxidized and that the formation of NiO occurs preferentially at grain edges, resulting from the reaction of the exsolved Ni particles with oxygen released from the perovskite lattice during decomposition via a spillover process from the perovskite to the Ni particles. Correlating electron microscopy and X-ray diffraction data allows us to establish a sequential two-step process in the decomposition of LaNiO_3_ via a Ruddlesden–Popper La_2_NiO_4_ intermediate structure. Exemplified for the archetypical LaNiO_3_ perovskite material, our results underscore the importance of focusing on both surface and bulk characterization for a thorough understanding of the catalyst dynamics and set the stage for a generalized concept in the understanding of state-of-the art catalyst materials on an atomic level.

## 1 Introduction

Understanding the physicochemical properties of heterogeneous catalysts is a prerequisite for establishing so-called “structure–activity/property” relationships, and it is therefore imperative for catalyst development and elucidating reaction mechanisms on an atomic level. As such, catalyst characterization is most powerful if conducted under real catalytic operating conditions, circumventing the pressure gap in catalysis.^1,2^ The latter is usually associated with the inability to correlate (ultra-) high-vacuum-based catalyst characterization with catalytic profiles obtained under technical catalytic conditions. *Insitu* spectroscopic^3^ or structural characterization^4,5^ of heterogeneous catalysts nowadays covers a range of sophisticated experimental methods, with electron microscopy techniques at the forefront. This is important insofar as the current understanding of the action of solid heterogeneous catalysts under catalytic operation is of a highly dynamic nature, where the catalyst surface and bulk quickly adapt themselves to the changes in the reaction environment.^4^ This encompasses activation or deactivation sequences, which go along with, *e.g*., reconstruction of surface and bulk structures,^6^ phase transformations,^7^ and redox reactions or exsolution phenomena.^8,9^ On the most detailed microscopic level, we strive to image the active sites and local dynamics relevant for an in-depth understanding of catalytic action.

In recent years, the use of precursor structures to prepare catalytically active and selective materials by deliberate and controlled decomposition in relevant catalytic reaction mixtures has gained increasing interest.^10−15^ A range of catalytic materials, including corrosion and self-activation of intermetallic compounds^11,12^ or deconstruction of perovskite structures^13,14^ have been thoroughly studied in a variety of reactions. Methanol steam reforming^16,17^ or methane dry reforming^18,19^ has been scrutinized. Especially for perovskite structures in reforming reactions, the concept of controlled decomposition is a particularly promising synthesis pathway to obtain highly dispersed metal particles in oxide matrices and the associated formation of a catalytically operating metal–oxide interface.^12,14,20,21^ This approach has been documented for a number of examples to result in superior materials in terms of activity and selectivity.^10,12,14^ Despite the several advantages of this method, one obstacle remains: perovskites are highly complex structures (even more so if they are doped with other elements) and are prone to a range of transformations before the catalytically active mixture is obtained.^22,23^ These transformations include, but are not limited to polymorphic transformations, formation of oxygen-deficient structures or transient structures. We emphasize that these structural changes go well beyond simple occurrence, but in fact, they play a very important role in the formation of the final active phase. We have documented this for a series of (doped) perovskite/Ruddlesden–Popper structures on La–Ni basis, especially LaNiO_3_ and La_2_NiO_4_, upon *insitu* treatment in a methane dry reforming mixture.^14,24^ The final structural fate is a Ni/La_2_O_3_/La_2_O_2_CO_3_ composite, but the road of decomposition involves a series of additional structural transformations into oxygen-deficient LaNiO_2.7_ or LaNiO_2.5_ and into La_2_NiO_4_, which appears as the most crucial structure before total deconstruction of the perovskite lattice. While the limited stability of the transient Ruddlesden–Popper structure La_2_NiO_4_ determines the onset of formation of the Ni/La_2_O_3_/La_2_O_2_CO_3_ interface, the loss of active oxygen and the associated oxidation of exsolved Ni to NiO during decomposition is of most importance. As long as oxygen can be delivered, NiO remains, and the interface is not catalytically active. As soon as the oxygen supply ceases and metallic Ni appears, the activity strongly increases.^12^

This work explicitly addresses the local and global structural and chemical dynamics upon decomposition of LaNiO_3_ by using analytical aberration-corrected *insitu* electron microscopy. To decouple thermal from chemical effects (those caused due to interaction with the reaction mixture) and to slow down the process of nickel exsolution and the associated structure evolution, we focus here on the decomposition of LaNiO_3_ under slightly reductive ultrahigh vacuum conditions. This allows step-by-step analysis of the individual decomposition and comprehending the role of reactive oxygen more closely. Consequently, we are able to transfer the results to the next level of complexity, i.e., more reducing conditions or the admission of a dry reforming mixture causing a rich carbon chemistry. To connect the local information to a global understanding of the catalyst structure, we use *insitu* synchrotron-based X-ray diffraction and *insitu* X-ray photoelectron spectroscopy for bulk and surface characterization of the Ni exsolution dynamics to pinpoint the temperature region of the first appearance of Ni at the surface more closely.

## 2 Experimental Procedures

### 2.1 Catalyst Synthesis

LaNiO_3_ was synthesized via a self-combustion method.^12,14^ Stoichiometric amounts of La(NO_3_)_3_ and Ni(NO_3_)_2_ were dissolved in water and mixed in an aqueous solution in a special modular beaker setup accounting for the safe performance and sample collection of the violent exothermal reaction (products). Glycine (NH_2_CH_2_CO_2_H) was dropwise added in an equimolar ratio NO_3_:NH_2_. The resulting greenish solution was then heated for approximately 3 h at 95 °C until the solution turned into viscous green gel. Upon further heating to 250 °C, the metal oxides, as well as carbon residues, were formed under a controlled exothermal reaction. Calcination at 750 °C for 10 h in air in a muffle furnace removed the remaining carbon with simultaneous formation of the rhombohedral LaNiO_3_ perovskite structure as evidenced by *ex situ* XRD.

### 2.2 *In Situ* Electron Microscopy

Transmission electron microscopy (TEM) analysis was performed on an FEI Titan 80–300 microscope with a C_s_-image corrector. Images were captured by a 2k × 2k Gatan UltraScan 1000 CCD camera. The operation accelerating voltage was 300 kV. Scanning transmission electron microscopy (STEM) experiments were conducted using an FEI Titan G2 80–200 microscope equipped with a C_s_-probe corrector and a HAADF detector. The microscope was operated at 200 kV and the probe semiangle was 24.7 mrad. Elemental maps were taken by energy-dispersive X-ray spectroscopy (EDX) using four large-solid-angle symmetrical Si drift detectors. Ni K-lines and La L-lines were used for the elemental map analysis. The quantification error is ±2 at. %. Electron energy loss spectrum (EELS) results were obtained with a postcolumn energy filter system (Enfinium ER 977, Gatan Inc., Pleasanton, CA, USA). O K-edge, Ni L_2,3_-edges, and La M_4,5_-edges were used to determine the elemental and phase distribution. In-situ heating experiments were performed using a MEMS-based heating holder system (Wildfire, DENSsolutions B.V., Delft, NL). We used a temperature profile, where the temperature was continuously increased from room temperature (RT = 23 °C) to 600 °C in steps of 50 °C and then heated to 800 °C in steps of 20 °C. At each temperature step, the specimen was held for 10–20 min.

### 2.3 *In Situ* X-ray Diffraction

The *in situ* high-temperature synchrotron XRD experiments in pure He were performed at the beamline 12.2.2 of the Advanced Light Source (ALS), Lawrence Berkeley National Laboratory, California.^25^ Diffraction patterns were collected in angle-dispersive transmission mode with a focused 25 keV monochromatic beam (λ = 0.4984 Å/15 μm spot size). The sample powder was heated in a 0.7 mm (inner diameter) quartz capillary under a continuous He gas flow (10 mL/min) injected through a 0.5 mm tungsten tube. The capillary was heated at a 10 °C/min heating rate to 800 °C in an infrared heated SiC tube furnace as described elsewhere.^26,27^ Diffraction patterns were recorded by a Pilatus3 S 1 M detector(981 × 1043 pixels, pixel size 172 × 172 μm^2^, 30 ms read-out time) every 60 s during the heating cycle. Rietveld refinement was performed using the FULLPROF program.^28^

### 2.4 *In Situ* X-ray Photoelectron Spectroscopy

The UHV system used for the near-ambient pressure X-ray photoelectron spectroscopy (NAP XPS) is a customized SPECS setup. Maintaining a base pressure below 10^–10^ mbar, it comprises an analysis chamber, which can be backfilled with oxidative, reductive and reactive gas atmospheres up to 30 mbar, a PHIOBOS 150 NAP hemispherical energy analyzer with an 1D-DLD detector, a μFOCUS 600 NAP monochromatic small spot X-ray source (Al Kα) and a Flood Gun (FG22/35). A complementary mass spectrometer allows the operando surface characterization. The system is used with a custom-made four-axes manipulator adapted for laser heating and gas-phase reactions. High resolution spectra of the Ni 2p/La 3d and Ni 3p region were collected in 50 °C steps from room temperature to 700 °C at a rate of 10 °C min^–1^. To perform a quantitative analysis of the surface-bound Ni species, we exclusively rely on the Ni 3p peak. The spectra were fitted with a Shirley-type background and the quantitative analysis based on the relative sensitivity factors (RSFs) [CasaXPS: Processing Software for XPS, AES, SIMS, and More, Casa Software Ltd.], as well as the different inelastic mean free paths using the predictive G1 formula according to Gries.^29^ Due to the small spin–orbit coupling of the Ni 3p peak, it was neglected in the fitting procedure

## 3 Results and Discussion

### 3.1 Dynamic Exolution of Ni and Structural Reconstruction of LaNiO_3_ during Vacuum Annealing

To investigate the dynamic exsolution of nickel and the structural reconstruction on LaNiO_3_, low magnification *insitu* TEM heating experiments were conducted in vacuo. The samples were stepwise heated from room temperature (RT = 23 °C) to 800 °C and subsequently recooled to RT. A representative image series of the structural evolution at different temperatures is shown in Figure S1. At room temperature, the sample shows strong diffraction and mass–thickness contrast on different areas of the sample due to its polycrystallinity and the presence of grain boundaries. Upon increasing the temperature to 600 °C, the strong contrast disappeared, inducing the structural evolution of LaNiO_3_. Starting at 700 °C and proceeding up to 800 °C, surface reconstruction of the LaNiO_3_ grains by roughening of the previously smooth grain edges was observed. The structural reconstruction happened quickly within 16.9 and 18.0 s at 700 and 750 °C (cf. Movie S1 and Movie S2). At this stage, dark-contrast particles formed on the initial LaNiO_3_ matrix mark the onset of the Ni exsolution process. After cooling to RT, the corresponding EDX map in Figure S2 confirms that the dark-contrast particles are indeed Ni-rich particles, evidencing the Ni exsolution during the vacuum heating process. Figure S3 shows an image sequence of the isothermal evolution of samples during Ni exsolution extracted from a supplementary movie file (Movie S3) at 800 °C. As a guide to the eye, the Ni exsolution process is marked representatively by red and yellow dashed circles in Figure S3.

**Figure 1:**
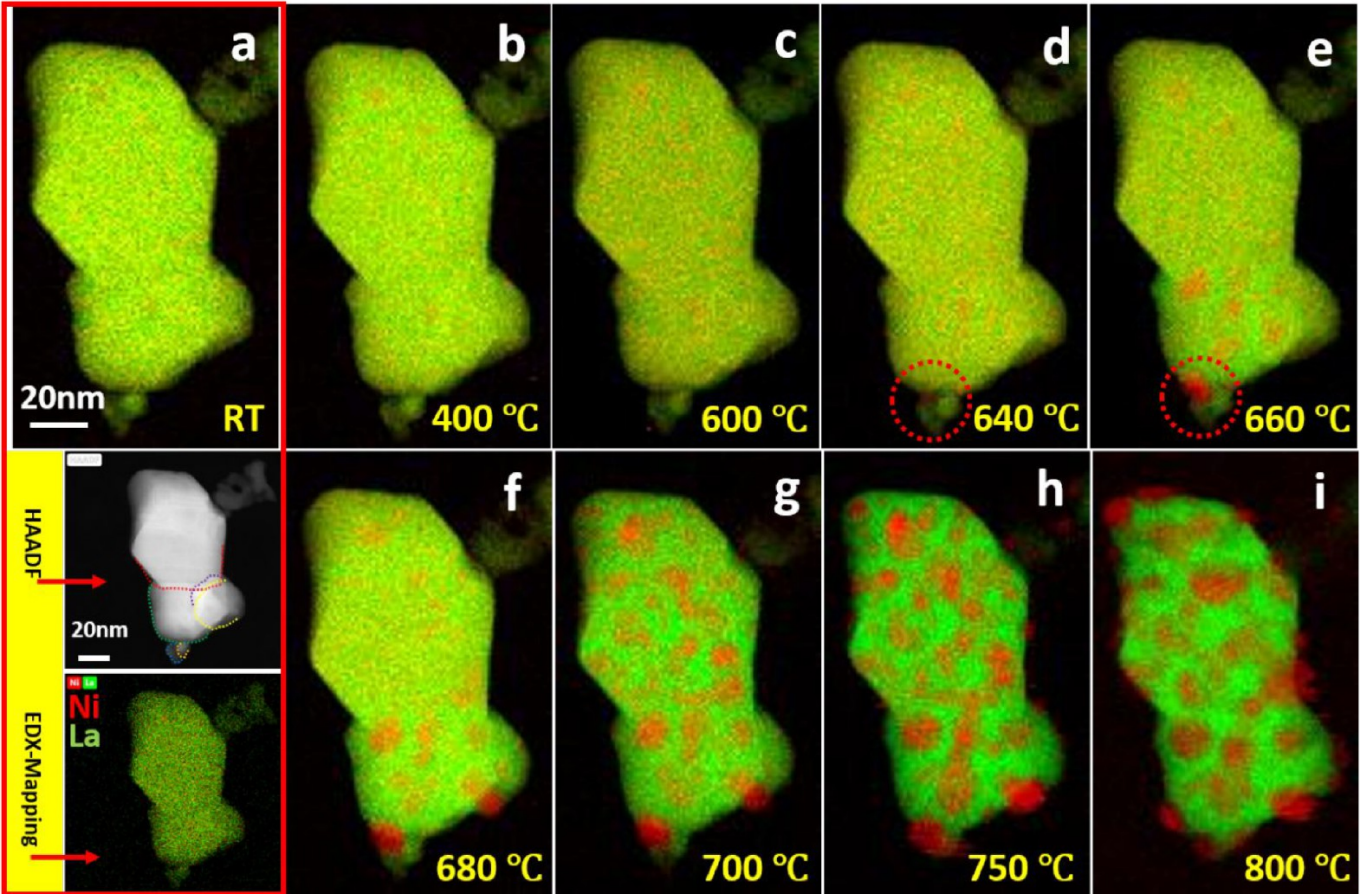
EELS maps of Ni and La distribution during the vacuum heating process of LaNiO_3_ at selected temperatures (a–i) using the MLLS fitting method. Green and red colors indicate La and Ni, respectively. Individual grains of LaNiO_3_ are marked by dashed lines in the inset HAADF image. In addition, an EDX map of the initial sample region is shown at the bottom of part a. Green and red colors indicate La and Ni, respectively.

In order to further probe the dynamic evolution of Ni species on LaNiO_3_ samples during vacuum heating, a sequence of *insitu* EELS spectrum-image elemental maps under the same heating process was captured and displayed in Figure 1. The EELS map data were fitted by the multiple linear least-squares (MLLS) method.^30^ As the initial structure of LaNiO_3_ is polycrystalline, the individual grains are outlined by dashed lines of different colors in the HAADF image in Figure 1a. Both the EELS and EDX map reveal that Ni and La are homogeneously distributed within the initial sample, i.e., at the beginning of the *insitu* heating experiment. As the temperature increases, local Ni element enrichment is observed at 600 °C, suggesting that the onset of detectable Ni exsolution occurs (Figure 1, parts b and c). Atomic diffusion of Ni toward the surface and the nucleation of Ni-rich particles occurs on the free surface upon heating to higher temperature.^21^ The formation of a Ni particle after heating from 640 to 660 °C is exemplified for the particles in the dashed red circles in Figure 1, parts d and e. Heating to a higher temperature led to the growth of this particle and the formation of new Ni particles. The nucleation/growth kinetics suggest that the Ni nucleation takes place at low temperatures, while its growth is favored at high temperatures.^31^ The initial formation of the Ni agglomerates or particles mainly occurs near grain boundaries at 660 and 680 °C, which indicates that the Ni exsolution process may prefer to transport and accumulate along the grain boundaries (Figure S4 and Figure 1d–f). At higher temperatures (700–800 °C), the formation of the Ni agglomerates is observed all over the grains (Figure 1g–i). The growth of Ni particles is faster on small grains than on large grains, as smaller grains decrease the elastic energy of the matrix.^32^ This result indicates that the grain size also affects the Ni exsolution process. In addition, the grain size of the remaining La-rich perovskite structure at 800 °C is smaller compared to the initial sample, which will be further discussed in connection with the results of the *insitu* HRTEM analysis. The probable explanation is that the Ni exsolution, which accompanies the decomposition of LaNiO_3_ into La_2_NiO_4_ and La_2_O_3_, compresses the crystal lattice.^33^

### 3.2 Role of Oxygen Dynamics during Decomposition of LaNiO_3_

**Figure 2:**
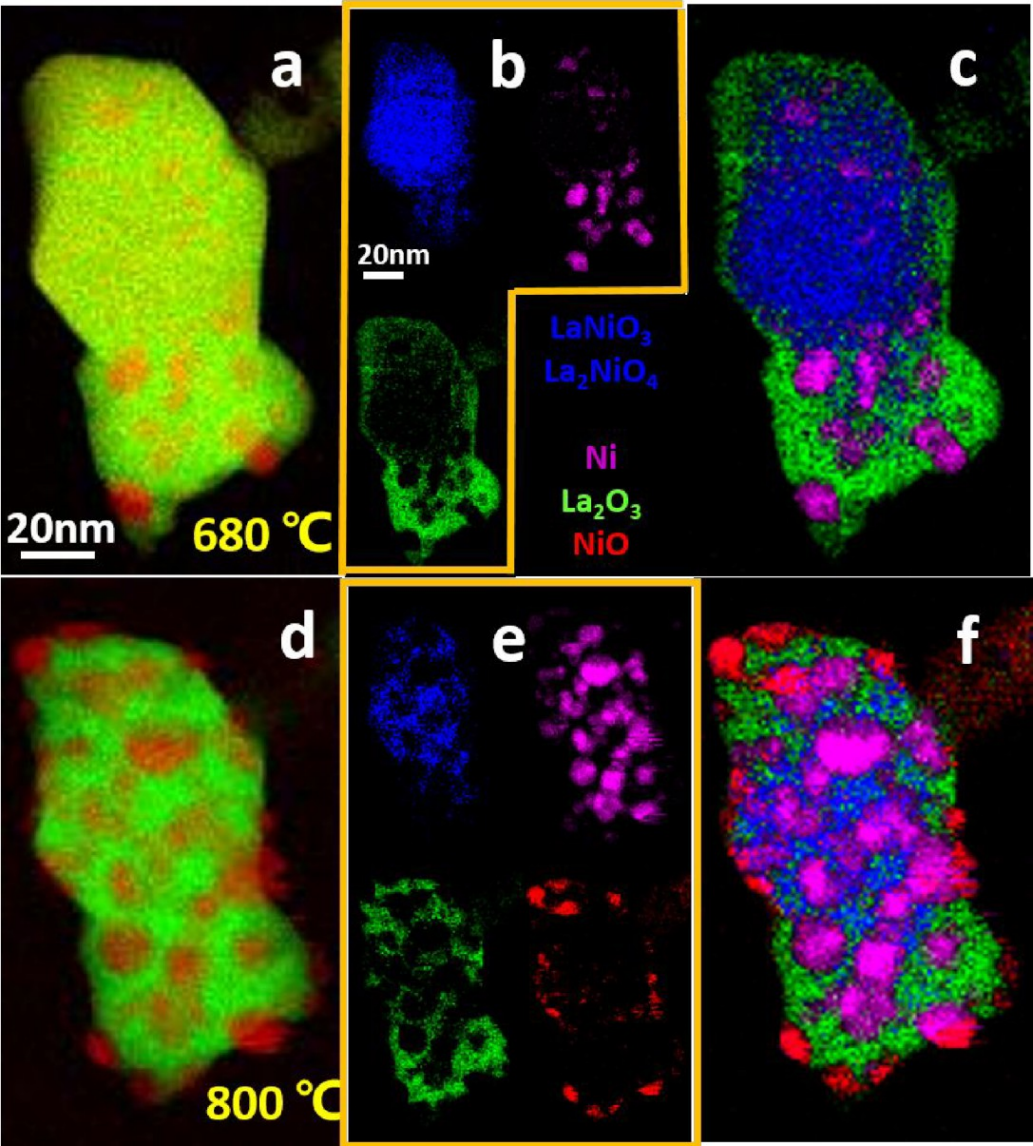
Comparison of an EELS map generated by the MLLS and SMA methods at 680 and 800 °C, i.e., at the onset of Ni exsolution and after progression of the exsolution process. Panels b and e display the independent components map using SMA, the phases LaNiO_3_/La_2_NiO_4_, Ni, La_2_O_3_ and NiO are indicated by blue, pink, green, and red colors. Panels c and f show the mixed components map using SMA.

On a global scale, the transformation of LaNiO_3_ during reduction and activation in the DRM mixture involves the oxygen-deficient LaNiO_2.7_ and LaNiO_2.5_ structures, as well as the Ruddlesden–Popper structure La_2_NiO_4_ en route to Ni/La_2_O_3_, has been proven by *insitu* XRD.^14,34^ It is therefore essential to investigate the local distribution of the individual components, especially oxygen, which is important for a thorough understanding of both their contribution to the Ni exsolution process and the catalytic action of LaNiO_3_. However, during the dynamic structure evolution process, it is a challenge to distinguish the spectra of LaNiO_3_ and La_2_NiO_4_ due to their similar EELS near-edge structure of the La, Ni, and O components.^35^ The superposition of different phases in the electron beam direction interferes with the unmixing of principal components. Here, multivariate statistical analysis (SMA)^36,37^ is applied to interpret and map the principal components from EELS spectrum data in more detail and, especially, to correlate with the *insitu* XRD experiments discussed below. The spectra are extracted and assigned to the corresponding components, as exemplified in Figure S5. Figure 2 shows the fitting results of the sample at representative temperatures of 680 °C (onset of Ni exsolution, Figure 2a–c) and 800 °C (Ni exsolution progressed, Figure 2d–f) using the MLLS and SMA methods. The Ni distribution map based on the MLLS method matches the Ni map generated by the SMA method. The overlap of different phases in the electron beam direction results in the presence of La and O signals in all principal components and a direct association with single phase is difficult. However, by our approach, we can attribute the principal components to the dominant phases. Accordingly, the principal component associated mainly with the spectrum of Ni is labeled as Ni, although La and O are present as well. At 800 °C, the distinction of different components reveals a clear difference between the SMA and the MLLS analysis. It is striking, that NiO is detected preferentially at the surface of the matrix (Figure 2f). Generally, along with Ni exsolution, oxygen loss occurs due to the decomposition of LaNiO_3_, creating oxygen vacancies.^38^ Under vacuum conditions, oxygen diffusion occurs from the LaNiO_3_ lattice to the surface, driven by the pressure gradient from the bulk to the sample surface.^39,40^ Thus, this surface oxygen likely oxidizes metallic Ni on the surface to NiO. Additionally, we also used EDX for quantification of the atom fractions of different areas (Figure S6 and Table S1). These results show, in accordance with our EELS-SMA results, that NiO is preferentially formed on the surface.

**Figure 3:**
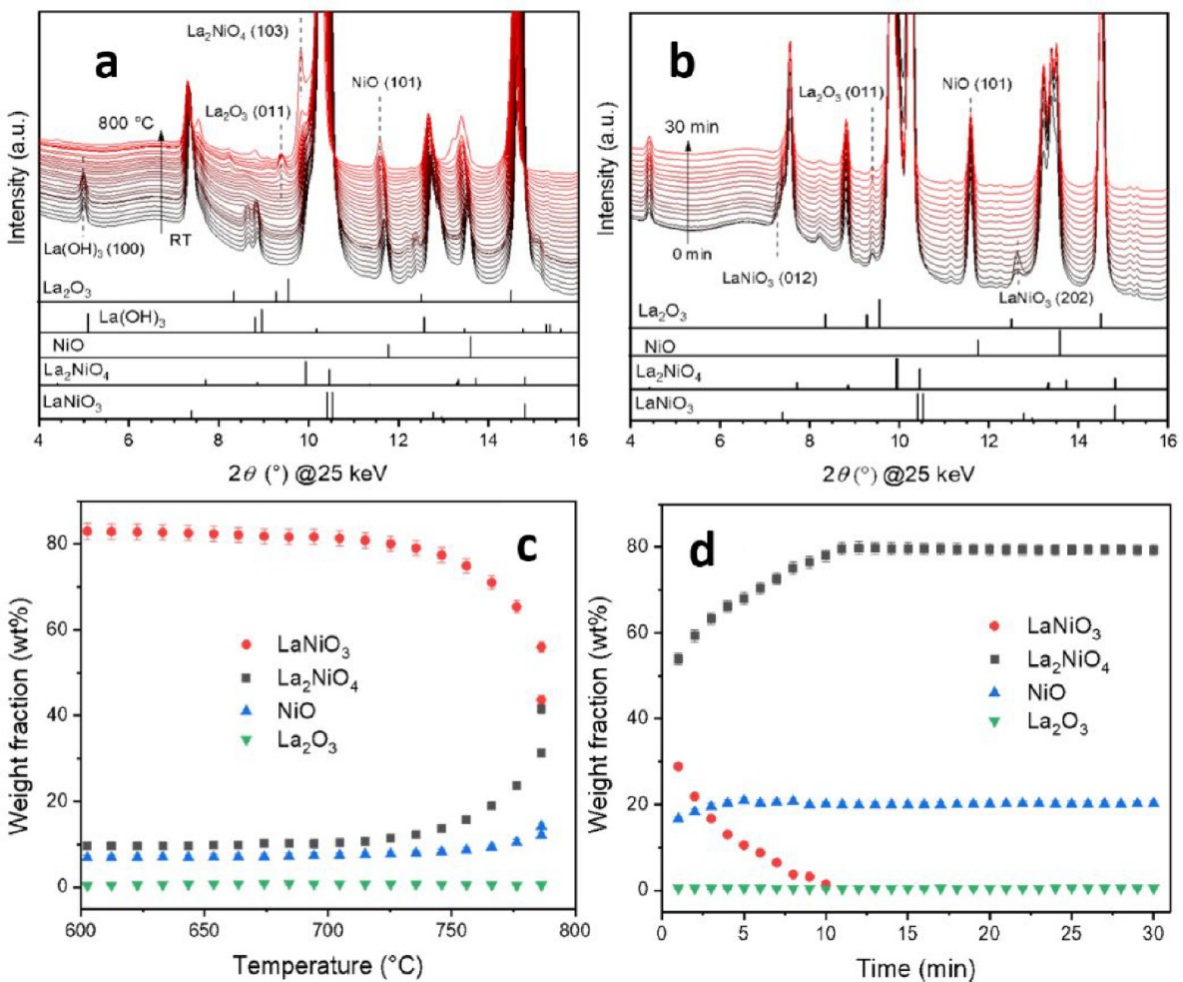
*Insitu* collected XRD patterns during heating LaNiO_3_ to 800 °C in He atmosphere. Panels a and b show the patterns during the heating and the isothermal part, respectively. The reference structures are shown as bars below the patterns. Panels c and d show the weight fraction analysis obtained from Rietveld refinement of the patterns above.

The formation of NiO was further confirmed by *insitu* X-ray diffraction during heating in He (Figure 3; full patterns over the entire collection angles shown in Figure S7). Figure 3a and b reveal, that LaNiO_3_ starts to transform at ∼760 °C into La_2_NiO_4_, which is completed after 10 min during the isothermal step at 800 °C. The amount of NiO is stable until ∼725 °C and later increases due to metallic Ni being oxidized by oxygen released during the transformation of LaNiO_3_ into La_2_NiO_4_ according to the reactions
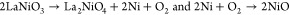
Rietveld refinement of the XRD patterns (Figure 3, parts c and d) reveals that the final phase composition of the sample after complete decomposition is 79.4 wt % La_2_NiO_4_, 20.1 wt % NiO, and 0.5 wt % La_2_O_3_ (Figure 3d). On a global scale, no other phases such as LaNiO_2.7_ and LaNiO_2.5_, which are present during hydrogen reduction and DRM, are observed during decomposition. To verify the bulk transformation equation of LaNiO_3_ into La_2_NiO_4_, we calculated the mole fractions of the crystalline phases (Figure S8). With increasing temperature and time, the increase in the mole fractions of both NiO and La_2_NiO_4_ are equal to half the decrease in the mole fraction of LaNiO_3_, confirming the bulk transformation of LaNiO_3_ into La_2_NiO_4_.

As expected, the thermal treatment induces partial decomposition of LaNiO_3_ and exsolution of Ni, but complete transformation into Ni/La_2_O_3_ is suppressed under the current experimental conditions. Most importantly, metallic Ni was not observed at any stage of the experiment. Corroborating the EELS-STEM results, also XRD reveals oxidation of Ni, resulting from the loss of reactive oxygen during decomposition of LaNiO_3_. As the *insitu* XRD was conducted under inert He, the pressure gradient from inside to the outside of the sample suppressed both further decomposition of La_2_NiO_4_ and oxygen diffusion, resulting in the reaction between lattice oxygen and exsolved Ni. Only the La_2_NiO_4_ and NiO components are detected at the end of the heating experiment. This result is of crucial importance for the understanding of the role of LaNiO_3_ self-activation during DRM, where the increase in activity was suggested to be linked with the transition of NiO to metallic Ni caused by the cessation of reactive oxygen supply from the decomposition of LaNiO_3_. Both *insitu* methods indicate that Ni oxidation is a direct result of oxygen transfer from the decomposing LaNiO_3_ to the exsolved Ni particles.

### 3.3 Atomic-Scale Insights into the Structural Evolution of LaNiO_3_ during Vacuum Annealing

High-resolution *insitu* TEM analysis was performed on LaNiO_3_ using the same temperature profile and under vacuum. The image series of *insitu* experiments on a LaNiO_3_ grain oriented along with the [0 0 1] and [2 4 1] zone axes at different temperatures are displayed in Figures S9 and S10, respectively. Based on the measured lattice spacings and the FFT pattern, the as-prepared LaNiO_3_ phase exhibits a rhombohedral structure (space group = *R*3*c*; lattice parameters *a* = 5.4573 nm, *b* = 5.4573 nm, and *c* = 13.1462 nm) at RT. The FFT and corresponding crystal structure of two representative samples were displayed in Figure S11. The crystal structure determination of as-prepared LaNiO_3_ is easier for crystals in [2 4 1] direction compared to the [0 0 1] direction, as the La and Ni atom columns are well separated in the orientation of [2 4 1]. Morphological changes were observed at the edges of both samples after heating to 640 °C. The smooth edges become rough at higher temperatures, which is attributed to the formation of La_2_O_3_ during the decomposition of LaNiO_3_, which is discussed and confirmed later. The more dramatic structural evolution occurred in the temperature range between 700 and 800 °C; i.e., the shape of samples is destroyed and reconstructed to particles with new shapes (Figure S9, parts f and h, and Figure S10, parts h and j).

**Figure 4:**
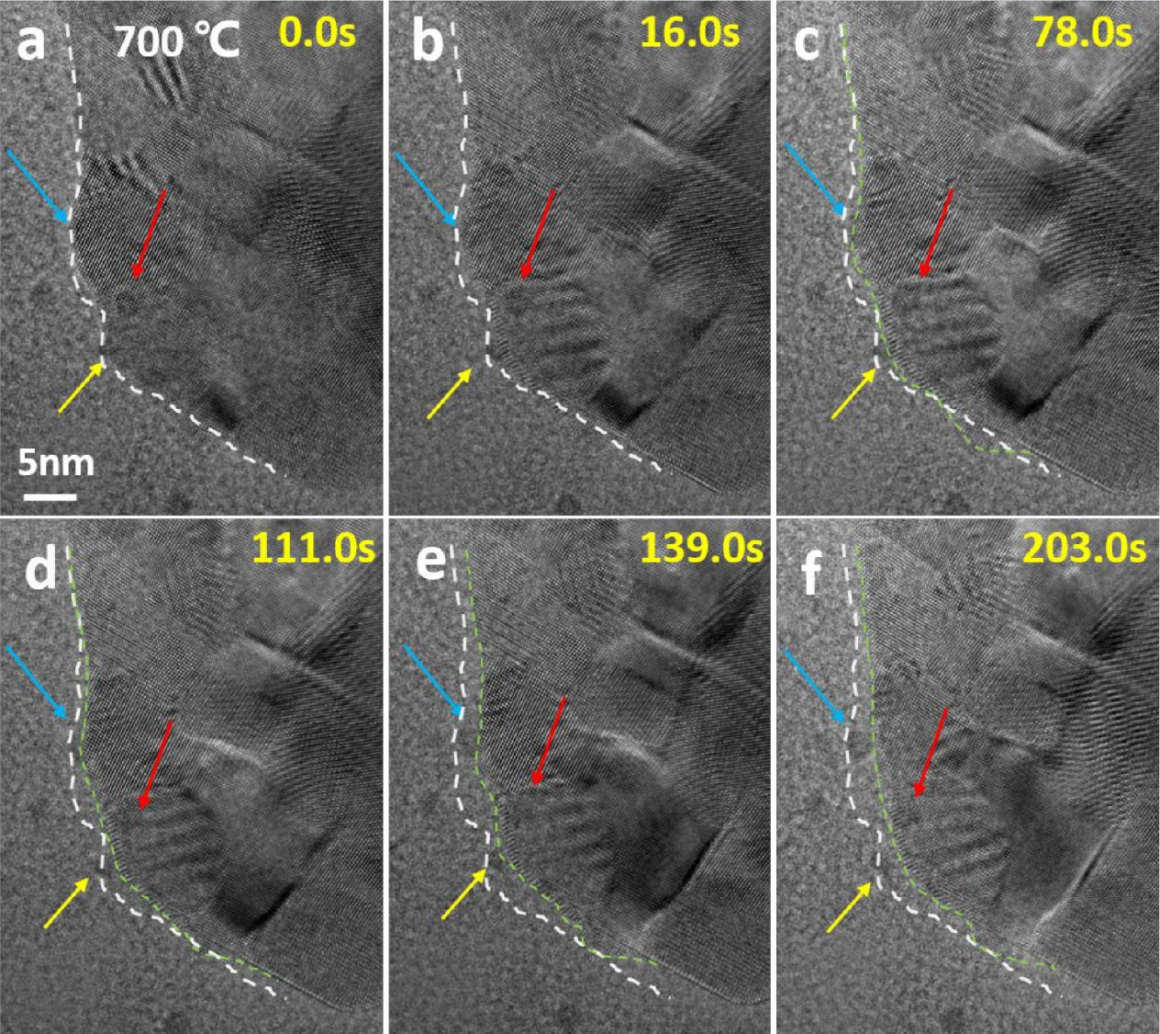
Direct observation of Ni particle detachment from reduced LaNiO_3_ at different times during an isothermal *insitu* vacuum heating step at 700 °C. Blue, red and yellow arrows highlight three different Ni particles. The white dashed lines indicate the initial edge shape of the sample and the green dashed lines represent the shape at different times during the isothermal heating experiment.

At 700 °C, the evolution of the structure was investigated over a duration of 203.0 s. The position of three representative Ni particles (marked by blue, red, and yellow arrows) were tracked in Figure 4. After a few seconds, the Ni particles start to diffuse on the La_2_O_3_ crystal and eventually completely detach from the perovskite grain. At the same time, the projected surface morphology of the sample underwent a significant change and a shrinking of the sample size was observed. Compared to the initial shape of the sample (outlined by white dashed lines), the shrinking process (marked by green dashed lines) was shown in Figure 4. As there is a large difference in lattice parameters for LaNiO_3_, La_2_NiO_4,_ and La_2_O_3_ phases, the structure evolution leads to the shrinking of the sample size during the vacuum heating process. Neagu et al.^21^ by providing insight into nanostructure-tailoring of Ni particles during the exsolution process from perovskites, proposed a Ni exsolution process involving nucleation, incipient socket, strong socking and socket relaxation steps. In line with their work, we suggest that the detachment of the Ni particles from the perovskite grain is due to the shrinking of the perovskite grain after the socket relaxation. It should be pointed out, that shrinking at high temperatures has a negative effect on the anchoring of Ni particles, which should be avoided during the reaction. The displayed Ni particle is strongly faceted in [101] direction at 650 °C, but its crystallinity is lost when the temperature was increased to 700 °C (Figure S10h, red dashed circles), implying that the structure of Ni particle is also undergoing dynamic changes. During *insitu* XRD heating, the transformation from LaNiO_3_ to La_2_NiO_4_ was observed at 760 °C. Based on our observations, at temperatures above 760 °C, the exsolved Ni particle can be quickly oxidized by diffused oxygen. Due to the very fast nature of the dynamic Ni exsolution process and the higher stability of embedded crystalline NiO^41^ at 760 °C, only NiO peaks are observed during *insitu* XRD experiments.

The shrinkage of the grains as a result of Ni exsolution was also examined by performing a quantitative analysis of the *insitu* X-ray diffraction patterns. The temperature dependence of lattice parameters *a* and *c* and unit cell volume of LaNiO_3_ perovskite is generally not linear in He atmosphere in the full temperature range (in Figures S12 and S13). The lattice parameters and unit cell volume exhibit a linear increase with temperature up to 650 °C, which is due to the thermal expansion effect. Above 650 °C, these parameters are rapidly increased, indicating the chemical expansion in the LaNiO_3_ perovskite. This chemical expansion can be attributed to the loss of oxygen from the perovskite lattice, which generates oxygen vacancies in the lattice and induces the reduction of smaller Ni^3+^ ions (0.56 Å) into larger Ni^2+^ ions (0.69Aring;) as the charge compensation.^42^ As a result, the lattice parameters and unit cell volume increase. These results suggest the formation of oxygen-deficient LaNiO_2.75_ and LaNiO_2.5_ structures above 650 °C. The transformation of LaNiO_3_ into LaNiO_2.75_ and LaNiO_2.5_ is accompanied by a volume variation, Δ*V*/*V*, of 1.65% and 6.38%, respectively.^43^ The volume variation determined from *insitu* XRD experiment at 800 °C (Figure S12c) was found to be about 1.25%, suggesting the formation of LaNiO_2.75_ phase. The slight difference between the value of Δ*V*/*V* determined at 800 °C in our experiments and the previously reported value at room temperature can be explained by the difference in the thermal expansion between both phases and/or the formation of fewer oxygen vacancies in the LaNiO_2.75_ phase. However, it is very difficult to confirm the formation of these oxygen-deficient materials according to the *insitu* XRD patterns due to the overlapping of their corresponding XRD reflections with those of LaNiO_3_, La_2_NiO_4_ and La_2_O_3_ phases presented in the sample.

Figure S12d shows the temperature dependence of crystallite size of LaNiO_3_ phase that was calculated by Rietveld refinement analysis. The crystallite size increases slightly with temperatures up to 400 °C, before increasing significantly in the temperature range 420–740 °C due to grain growth. Above 740 °C, a remarkable decrease in the crystallite size of LaNiO_3_, which is accompanied by the partial transformation of LaNiO_3_ into La_2_NiO_4_ and NiO phases. These results are consistent with the TEM characterization (Figure 4), which reveals the shrinkage of LaNiO_3_ grains during the transformation and exsolution of Ni from its lattice.

**Figure 5:**
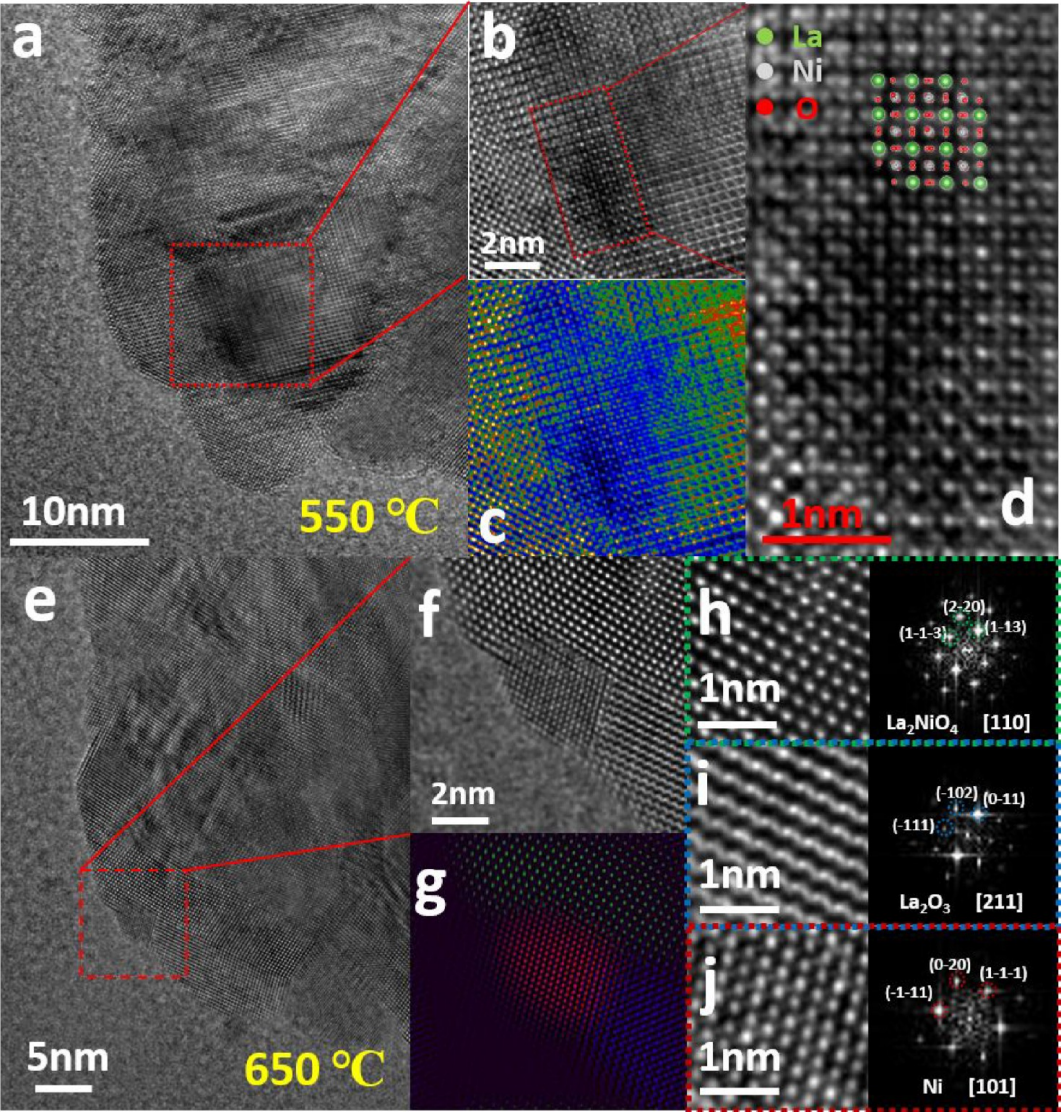
(a) NCSI High-resolution TEM image of the sample at 550 °C, (b) cropped dark area near the grain boundary, (c) intensity profile of the cropped area, and (d) crystallographic structure matching to atomic-scale TEM image wherein the green, white, and red dots are corresponding to La, Ni, and O atoms. (e) NCSI high-resolution TEM image of the sample at 650 °C, (f) cropped image where the coexistence of La_2_NiO_4_, Ni, and La_2_O_3_ is shown, (g) the colored structure map of cropped HRTEM image wherein the green, red, and blue colors indicate La_2_NiO_2_, Ni, and La_2_O_3_, respectively, and (h–j) the corresponding FFTs of the images of La_2_NiO_4_, La_2_O_3_, and Ni.

To further investigate the mechanism of Ni exsolution, atomic-scale HRTEM investigations on Ni exsolution in LaNiO_3_ under negative spherical aberration imaging (NCSI) conditions^44,45^ were conducted on a particle in the [2 4 1] zone axis orientation. In this case, the stacking faults and were observed on the LaNiO_3_ (in Figure S11a) at the initial stage, where the stacking faults are formed in rhombohedral LaNiO_3_ during the calcination process in air.^46^ It is found that heating to 550 °C does not lead to significant morphology changes. However, compared to the same position of the sample at RT where the intensity contrast is uniform, areas near the stacking faults and grain boundaries became darker (in Figure 5a, enlarged in Figure 5b). The corresponding intensity profile (Figure 5c) shows significant NCSI contrast of Figure 5b, the brighter, the more atoms in columns. The NCSI method offers a way to directly image O atom columns in TEM mode.^47^ As shown in Figure 5d, the atomic resolution HRTEM image of LaNiO_3_ is matching well the standard crystallographic structure (zone axis [2 4 1]), where it is possible to pinpoint the location of all elements. Compared to the standard crystallographic structure, the upper part exhibits the perfect atomic structure of LaNiO_3_ where the Ni and O atoms columns are fully filled. In contrast, the lower part is darker than the upper part, where parts of Ni and O atoms are found to be absent. This is direct evidence to prove the atomic exsolution of Ni and the O release in LaNiO_3_ under vacuum heating. The structural evolution of LaNiO_3_ occurs at 550 °C where the Ni and O atoms exsolved from the lattice. As a result, LaNiO_3_ starts to convert to a intermediate LaNi_1–*x*_O_3-y_ phase. In the temperature range beween 550 and 600 °C, the exsolved Ni atoms and small clusters on the surface are too small to be directly imaged. Therefore, it indicates that the Ni exsolution prefers starting near stacking faults and grain boundaries on LaNiO_3_ and then spreads across the whole sample, which is consistent with *insitu* EELS analysis. Based on the *insitu* XRD results, the observation of a gradual transformation from LaNiO_3_ to LaNi_1–*x*_O_3-*y*_ suggests that LaNiO_3_ first decomposes into La_2_NiO_4_ at the beginning of the Ni exsolution process under vacuum heating conditions. Heating up to 650 °C, Ni particles oriented along with the [1 0 1] zone axis are detected at atomic-scale (Figure 5, parts e and f), directly confirming the existence of metallic Ni particles. FFTs of the enlarged HRTEM images demonstrate the coexistence of La_2_NiO_4_, Ni, and La_2_O_3_ (Figure 5g–j). This result indicates that La_2_NiO_4_ takes place a deeper decomposition to form Ni and La_2_O_3_.

To correlate the bulk dynamics of LaNiO_3_ during heating in vacuo in the electron microscope and under inert atmosphere via synchrotron-based X-ray diffractometry to surface elemental changes and to pinpoint the appearance of Ni at the surface more closely, we performed an *insitu* X-ray photoelectron spectroscopy analysis during heating in 0.2 mbar H_2_ between room temperature and 700 °C (Figure S14). We opted to perform the heating under a reducing background pressure to induce the Ni exsolution and to more clearly follow the onset of Ni exsolution. It indicates that surface-bound metallic Ni is first observed at 300 °C by its Ni 2p_3/2_ component at 852.6 eV binding energy. As expected, the amount of metallic Ni increases upon heating to 700 °C. The four strong peaks featuring the well-separated spin–orbit coupling result from only one La component. At 300 °C, the La 3d_5/2_ region by shape and multiplet splitting is characteristic of the presence of surface carbonates resulting from adsorption of carbon-containing species. A gradual transition to a spectral fingerprint of La_2_O_3_ at 700 °C is observed. This is in line with both the *insitu* TEM and XRD analysis, showing the presence of La_2_O_3_ after partial decomposition. The *insitu* XPS measurements show that in fact, Ni exsolution starts and proceeds at much lower temperatures than anticipated from the appearance as nm-sized particles in TEM or crystallized particles in XRD. The more reducing the environment, the more difficult the monitoring of the formation of NiO as controlled by oxygen diffusion. The TEM experiments (corroborated by the *insitu* XRD experiments on a global scale), due to their only slightly reducing conditions, provide the most accurate experimental setting to monitor the oxidation of Ni by oxygen released from the decomposition of LaNiO_3_.

**Figure 6:**
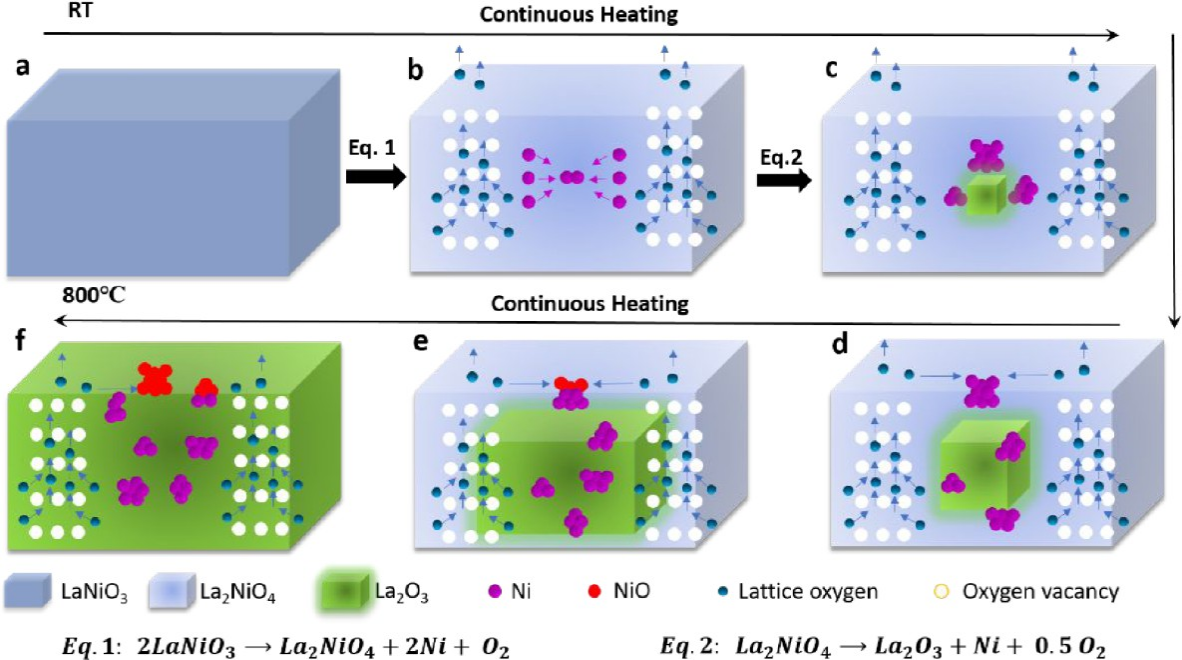
Schematic mechanism for structural evolution of LaNiO_3_, Ni exsolution, and oxygen diffusion during vacuum annealing.

The mechanism for the structural evolution of LaNiO_3_, specifically for Ni exsolution and oxygen diffusion, during the vacuum heating is schematically displayed in Figure 6. Based on *insitu* HRTEM, EELS and XRD results, it can be concluded that the Ni exsolution process in vacuo proceeds via two sequential steps. LaNiO_3_ is first decomposed into La_2_NiO_4_ (eq 1 in Figure 6b), which is then converted to La_2_O_3_ during further Ni exsolution (eq 2 in Figure 6c). Ni exsolution undergoes the stages of atom exsolution, nucleation, embedding, half-embeddeding, and eventual detachment during vacuum heating. Accompanying the Ni exsolution, oxygen vacancies are formed and lattice oxygen is released under reduction conditions. Our results indicate that the formation of NiO results from the reaction of the exsolved Ni particles with surface oxygen released from the perovskite lattice during decomposition via a spillover process from the perovskite to the Ni particles (Figure 6, parts d and e).

## 4 Conclusions

We provide atomic insight into the complex surface and bulk catalyst structure evolution of a LaNiO_3_ perovskite material under vacuum heating conditions to follow the transition from the perovskite over the transient La_2_NiO_4_ structure into a Ni/La_2_O_3_ composite. The vacuum heating experiments provide us with the opportunity to slow down the exsolution process of small Ni particles and the associated structural breakdown of the parent LaNiO_3_ material to monitor the structural and chemical changes more closely. The changes include the predominant exsolution of Ni nanoparticles from grain edges and stacking faults in combination with a drastic shrinkage of the grain volume of the parent LaNiO_3_ during the exsolution process. Structural changes were also assessed on a local scale on the basis of EELS mapping experiments, allowing us to map the distribution and evolution of different phases at different stages of the exsolution process. Important for catalytic applications, we provide evidence for the spillover of reactive lattice oxygen from the decomposing LaNiO_3_ structure to the exsolved Ni particles, forming NiO.

The results represent a first step to the full understanding of the LaNiO_3_ material under catalytic operation. LaNiO_3_ is a highly active methane dry reforming catalyst, which undergoes significant structural and chemical changes in the self-activation step during reaction. As the reactive mixture includes both carbon dioxide and methane, simplification and deconstruction of the individual reaction steps are necessary. During the dry reforming reaction, thermal effects, reduction, and adsorption effects are superimposed and are very difficult to disentangle. Through deliberately suppressing strong reduction and reactant adsorption effects by vacuum annealing, our approach provides a simplistic, yet very important first step in the understanding of the local and global structure and chemical alterations of a complex catalyst material.

## Supporting Information Available

Low magnification images of an *in**situ* TEM heating experiment on LaNiO_3_ at selected temperatures under bright-field conditions, HAADF image and EDX map of LaNiO_3_ after the full heating–cooling cycle with a maximum temperature of 800 °C, bright-field TEM image sequence of the time evolution showing the Ni exsolution, HAADF image of LaNiO_3_ and EELS map of Ni and La distribution at 660 °C, extracted distinct spectra for LaNiO_3_/La_2_NiO_4_, Ni, La_2_O_3_, and NiO components using the SMA method, EDX quantification in atomic fraction for the representative areas, *in**situ* collected XRD patterns of LaNiO_3_ during heating to 800 °C in He atmosphere and during an isothermal period for 30 min in He atmosphere, mole fractions of different crystalline phases formed during heating LaNiO_3_ up to 800 °C for 30 min in He atmosphere as a function of temperature, a series of high-resolution TEM images of LaNiO_3_ in [001] orientation during the vacuum heating experiment, Series of high-resolution TEM images of LaNiO_3_ in [241] orientation during the vacuum heating experiment, evolution of the lattice parameters *a* and *c* and unit cell volume and the crystal size of LaNiO_3_ samples as a function of reaction temperature, XRD reflections corresponding to the (024) lattice planes of the LaNiO_3_ phase at 715 and 775 °C, initial room-temperature structure of LaNiO_3_ imaged in two different crystallographic orientations at room temperature, *in**situ* X-ray photoelectron spectroscopy analysis of the Ni 2p and La 3d region for heating LaNiO_3_ in 0.2 mbar H_2_ from room temperature to 750 °C (PDF)
Movie S1, dynamic structure evolution of LaNiO_3_ at 700 °C (AVI)
Movie S2, dynamic structure evolution of LaNiO_3_ at 750 °C (AVI)
Movie S3, dynamic structure evolution of LaNiO_3_ at 800 °C (AVI)

## Supplementary Material

jp1c09257_si_001.pdf

jp1c09257_si_002.avi

jp1c09257_si_003.avi

jp1c09257_si_004.avi
