# Supplementary material for: Atomic-Scale Insights into Nickel Exsolution on LaNiO3 Catalysts via In Situ Electron Microscopy
Source: J Phys Chem C Nanomater Interfaces. 2021 Dec 30;126(1):786–96. doi: 10.1021/acs.jpcc.1c09257 (PMC8762657; doi:10.1021/acs.jpcc.1c09257)
Supplement: Supplementary file 1 — jp1c09257_si_001.pdf [file jp1c09257_si_001.pdf]

# Supporting Information:

# Atomic-scale Insights into Ni Exsolution on $LaNiO_3$ Catalysts via in situ Electron Microscopy

Pengfei Cao[a,b #], Pengyi Tang[b,e #], Maged F. Bekheet[d], Hongchu Du[b], Luyan Yang[2b], Leander Haug[c], Albert Gili[d], Benjamin Bischoff[d], Aleksander Gurlo[d], Martin Kunz[e], Rafal E. Dunin-Borkowski[b], Simon Penner[c], Marc Heggen[b*]

[a] *School of Chemical Engineering and Technology, Xi'an Jiaotong University, Xi'an 710049, China.*

[b] *Ernst Ruska-Centre for Microscopy and Spectroscopy with Electrons, Forschungszentrum Jülich GmbH, Leo-Brandt-Str. 1, D-52428 Jülich, Germany*

[c] *Department of Physical Chemistry, University of Innsbruck, Innrain 52c, A-6020 Innsbruck*

[d] *Chair of Advanced Ceramic Materials, Institut für Werkstoffwissenschaften und -technologien, Technical University Berlin, Hardenbergstr. 40, D-10623 Berlin, Germany*

[e] *Advanced Light Source, Lawrence Berkeley National Laboratory, Berkeley, California 94720, USA*

*Email: m.heggen@fz-juelich.de

*#P.C. and P.T. contributed equally to this paper*

Keywords:

Perovskites, in situ TEM heating, in situ X-ray diffraction, in situ X-ray photoelectron spectroscopy, electron-energy loss spectroscopy

**Results and Discussion**

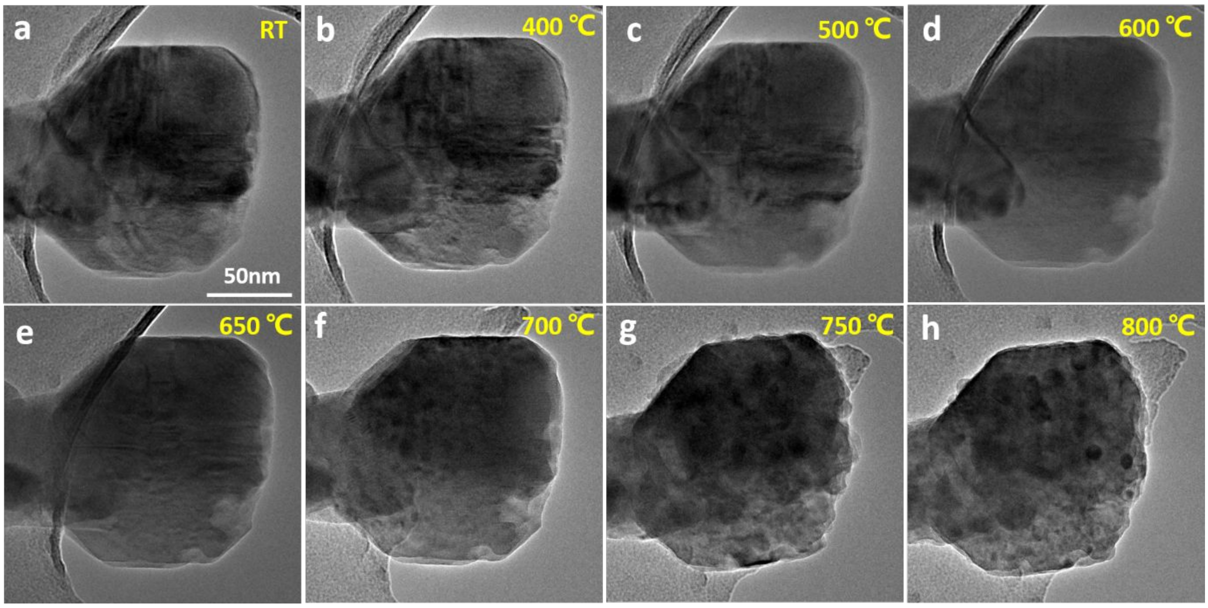


**Figure S1**(a)-(h): Low magnification images of an in-situ TEM heating experiment on $LaNiO_3$ at selected temperatures under bright-field conditions.

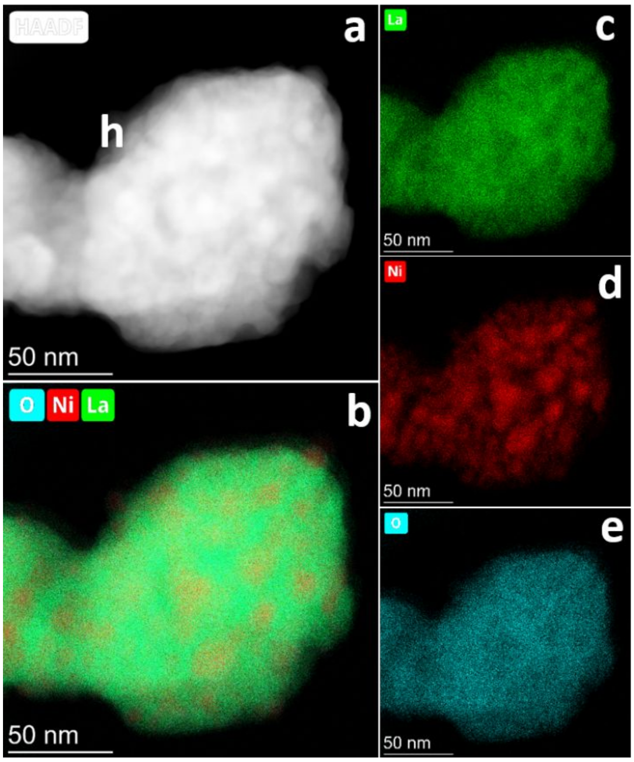


**Figure S2:** HAADF image and EDX map of $LaNiO_3$ after the full heating-cooling cycle with a maximum temperature of 800 °C. (a) HAADF images; (b) map depicting the superposition

Ni-K, La-L and O-K intensities; Independent elemental distribution maps of Ni-K, La-L, and O-K are shown in (c)-(e).

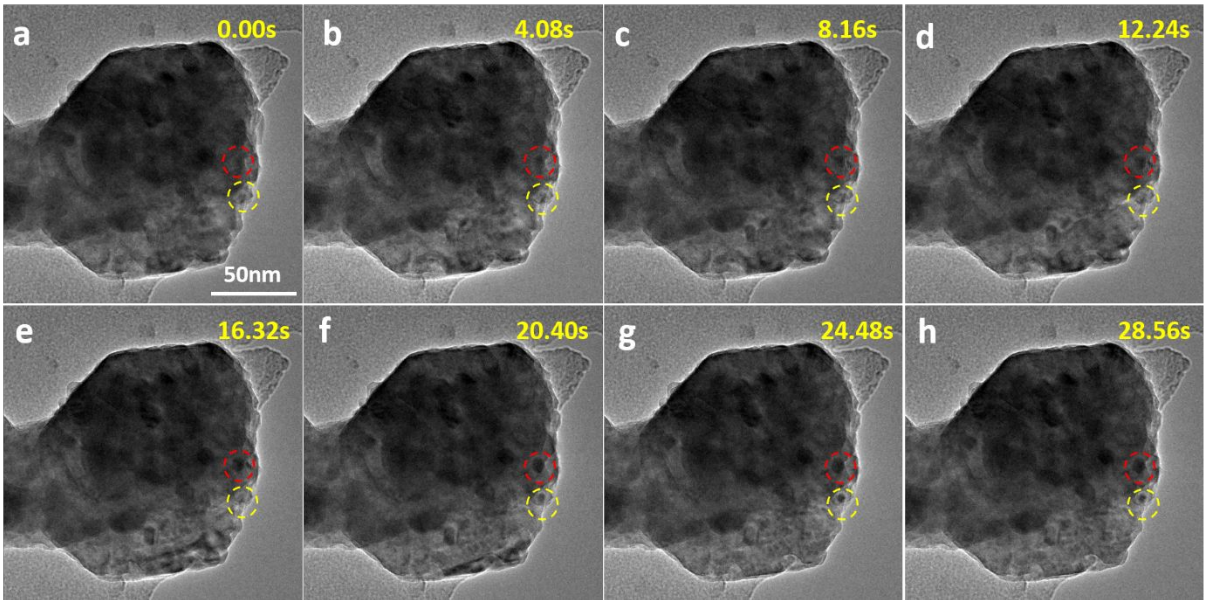


**Figure S3** Bright-field TEM image sequence of the time evolution showing the Ni exsolution (from supplementary movie file 3, a-h) during an in-situ heating experiment on $LaNiO_3$ under vacuum at 800°C. Red and yellow dash circles mark representative areas, where Ni exsolution occurs.

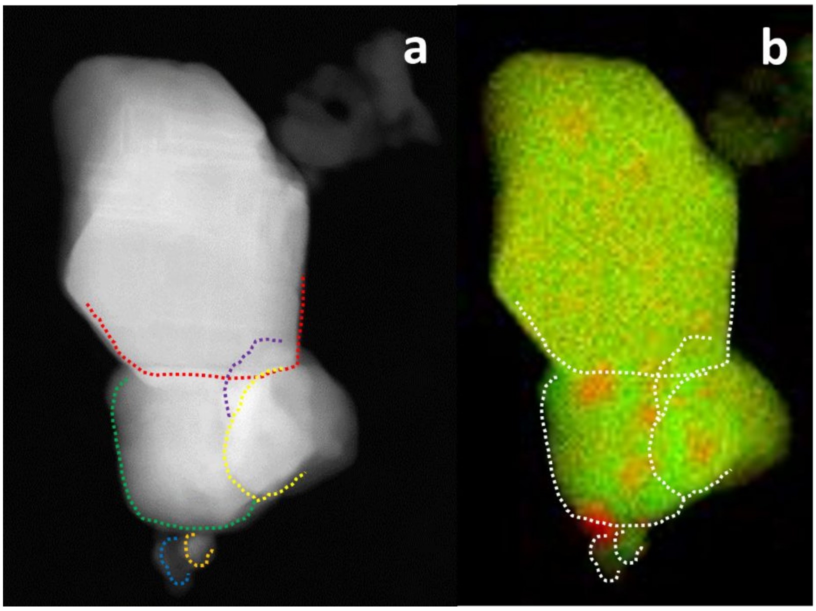


**Figure S4** (a) HAADF image of $LaNiO_3$, (b) EELS map of Ni and La distribution at 660°C, Green and red color indicate La and Ni, respectively. Individual grains of $LaNiO_3$ are marked by dashed lines. Ni agglomerates are mainly located near grain boundaries.

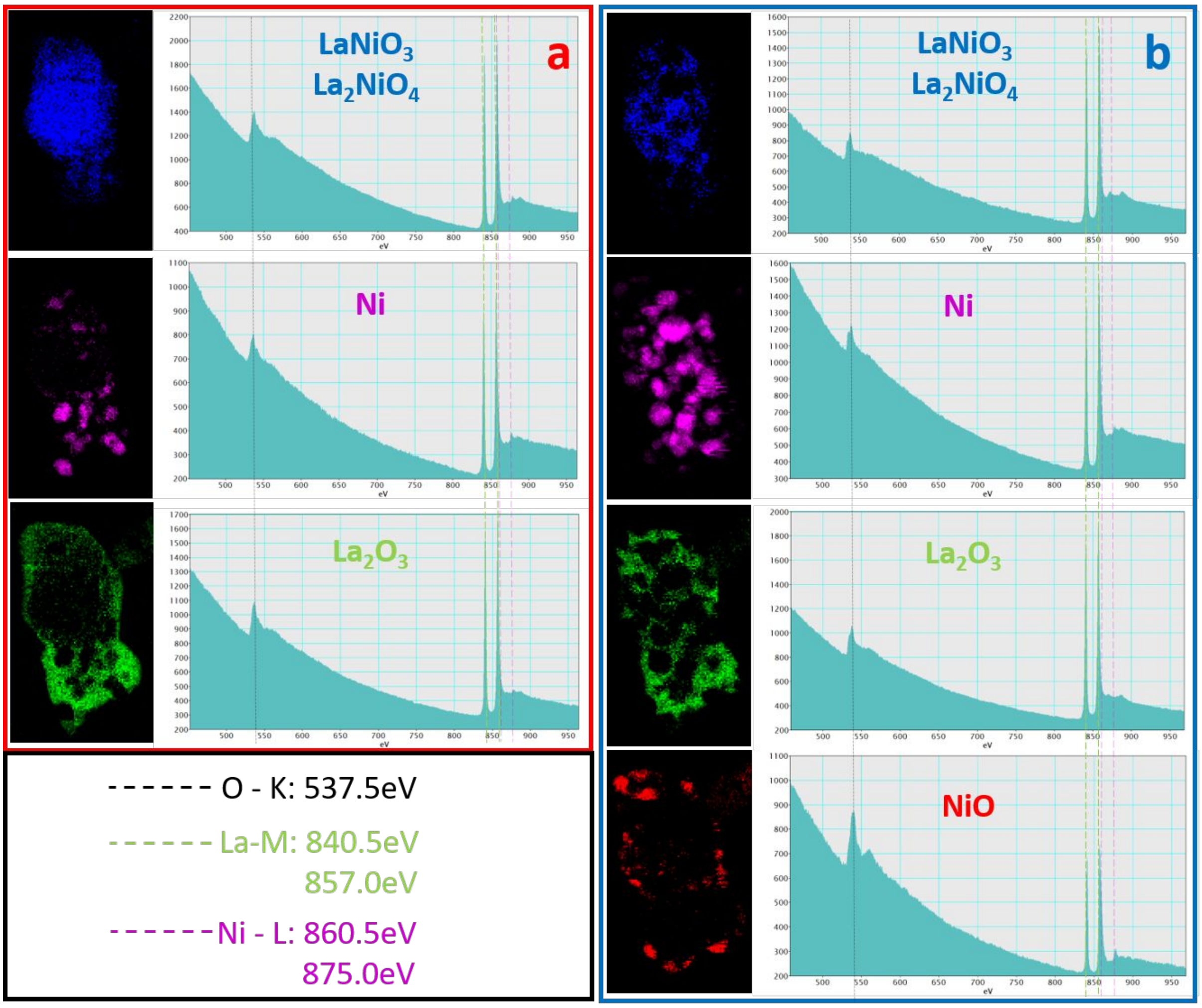


**Figure S5** Extracted distinct spectra for $LaNiO_3/La_2NiO_4$, Ni, $La_2O_3$, and NiO components using the SMA method. (a) 680 °C, (b) 800 °C.

In Figure S5, we defined the principal components based on the intensity changes of the EELS near-edge signal of La-M, Ni-L and O-K. For the extracted Ni spectrum, the intensity of the Ni-L signal becomes stronger while the O-K and La-M signals show a significant decrease compared to the spectra of $LaNiO_3/La_2NiO_4$, implying the local enrichment of Ni. The existence of O-K and La-M signals is due to their superposition in the electron beam direction on the sample. Obtained by a similar analysis, the extracted NiO spectrum reveals a lower intensity of La-M signal but higher intensities of the Ni-L and O-K signals as compared to the extracted Ni spectra. For the extracted $La_2O_3$ spectrum, it is defined that the spectrum shows

very weak Ni-L intensity, but strong La-M intensity compared to the spectrum of $LaNiO_3/La_2NiO_4$. The EELS results are supported by an EDX analysis (Figure S6 and Table1). As shown in Figure S6, Ni and O enrichment is found at the edge of the La-rich particle (site 1), but no La is detected at the same location. The particles in the center of the particle are generally less rich in NiO. This confirms that NiO is formed at the edges after vacuum heating.

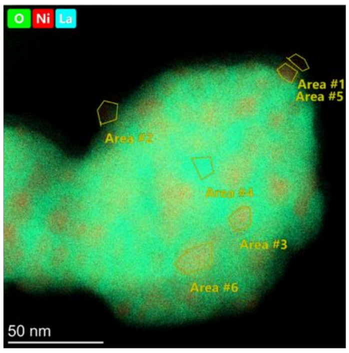


Analysis of spectrum: Spectra from Area #1

| Z | Element | Family | Atomic Fraction(%) | Atomic Error(%) |
|---|---|---|---|---|
| 8 | O | K | 33.82 | 5.97 |
| 28 | Ni | K | 60.74 | 12.96 |
| 57 | La | L | 5.43 | 1.13 |

Analysis of spectrum: Spectra from Area #2

| Z | Element | Family | Atomic Fraction(%) | Atomic Error(%) |
|---|---|---|---|---|
| 8 | O | K | 51.79 | 6.31 |
| 28 | Ni | K | 37.82 | 6.75 |
| 57 | La | L | 10.39 | 1.67 |

Analysis of spectrum: Spectra from Area #4

| Z | Element | Family | Atomic Fraction(%) | Atomic Error(%) |
|---|---|---|---|---|
| 8 | O | K | 59.47 | 7.07 |
| 28 | Ni | K | 10.14 | 1.81 |
| 57 | La | L | 30.4 | 4.88 |

Analysis of spectrum: Spectra from Area #3

| Z | Element | Family | Atomic Fraction(%) | Atomic Error(%) |
|---|---|---|---|---|
| 8 | O | K | 40.47 | 4.89 |
| 28 | Ni | K | 38.8 | 6.96 |
| 57 | La | L | 20.45 | 3.30 |

Analysis of spectrum: Spectra from Area #5

| Z | Element | Family | Atomic Fraction(%) | Atomic Error(%) |
|---|---|---|---|---|
| 8 | O | K | 36.2 | 4.96 |
| 28 | Ni | K | 50.86 | 9.69 |
| 57 | La | L | 12.94 | 2.25 |

Analysis of spectrum: Spectra from Area #6

| Z | Element | Family | Atomic Fraction(%) | Atomic Error(%) |
|---|---|---|---|---|
| 8 | O | K | 39.95 | 4.83 |
| 28 | Ni | K | 39.83 | 7.17 |
| 57 | La | L | 20.22 | 3.28 |

**Figure S6** EDX quantification in atomic fraction for the representative areas.

**Table S1** The analysis of the principal components by an atoms balance calculation for selected areas. The calculations are based on the results of the EDX quantification

| Position | $LaNiO_3$ (mol%) | $La_2NiO_4$(mol%) | $La_2O_3$(mol%) | NiO(mol%) | Ni(mol%) |
|---|---|---|---|---|---|
| Site 1 | 0 | 0 | 4.23 | 40.70 | 55.07 |
| Site 2 | 0 | 0 | 12.08 | 87.92 | |
| Site 3 | 0 | 0 | 20.86 | 20.40 | 58.74 |
| Site 4 | 31.95 | 24.89 | 43.16 | 0 | 0 |
| Site 5 | 0 | 0 | 11.29 | 29.29 | 59.42 |
| Site 6 | 0 | 0 | 20.25 | 16.80 | 62.95 |

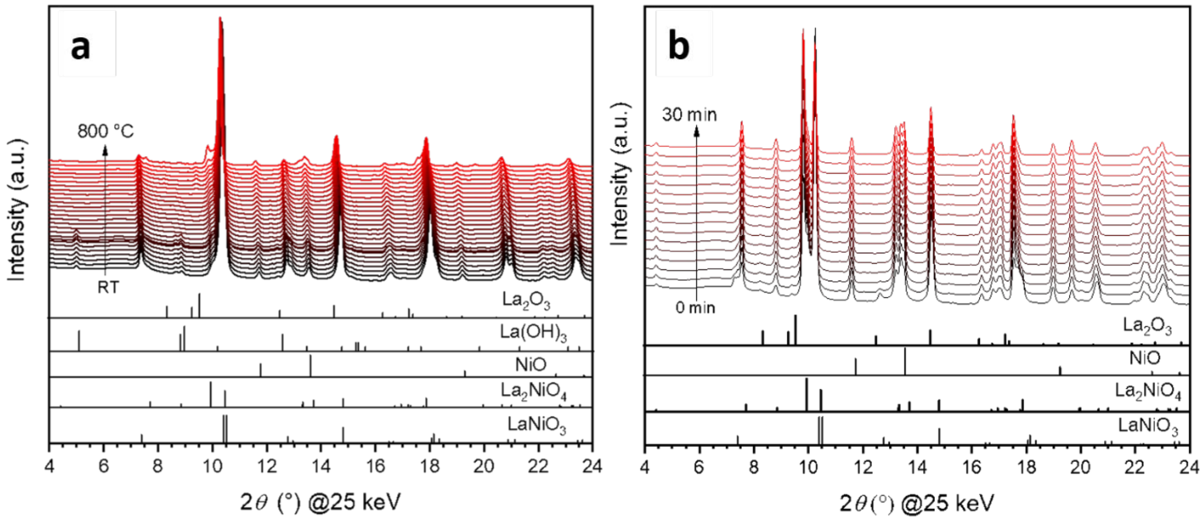


**Figure S7** (a) In situ collected XRD patterns of $LaNiO_3$ during heating up to 800 °C in He atmosphere; (b) In situ collected XRD patterns of $LaNiO_3$ during an isothermal period for 30 min in He atmosphere; The lower panels indicate the assignment to the respective reference structures.

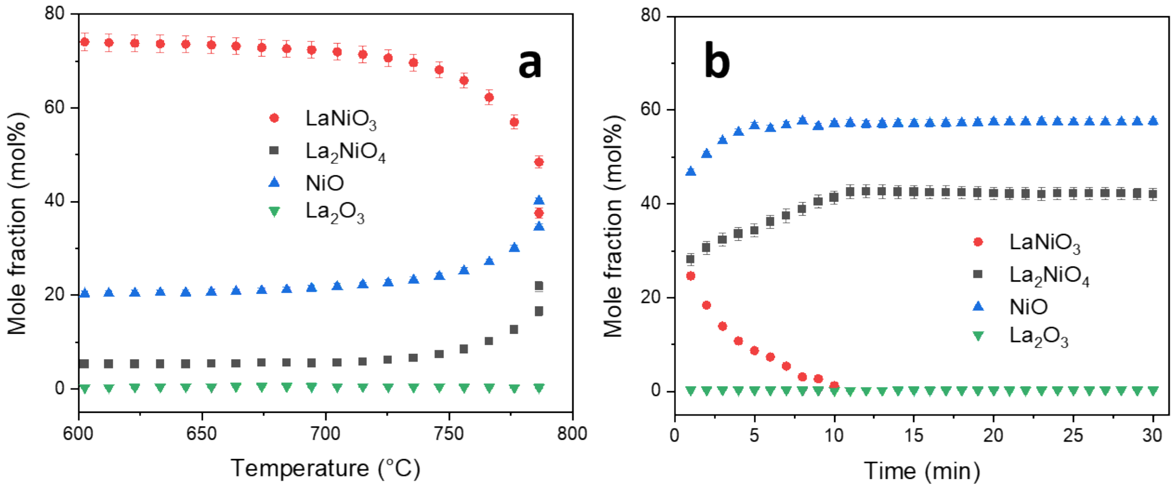


**Figure S8** Mole fractions of different crystalline phases formed during heating $LaNiO_3$ up to 800 °C for 30 min in He atmosphere as a function of temperature (a) and time at 800 °C (b) obtained by Rietveld refinement of the in situ collected XRD patterns. The mol% of these crystalline phases by dividing the weight fraction of each phase by its molar mass $x_i$ (mol%)= $(wt_i/M_i)/sum_k (wt_k/M_k)$.

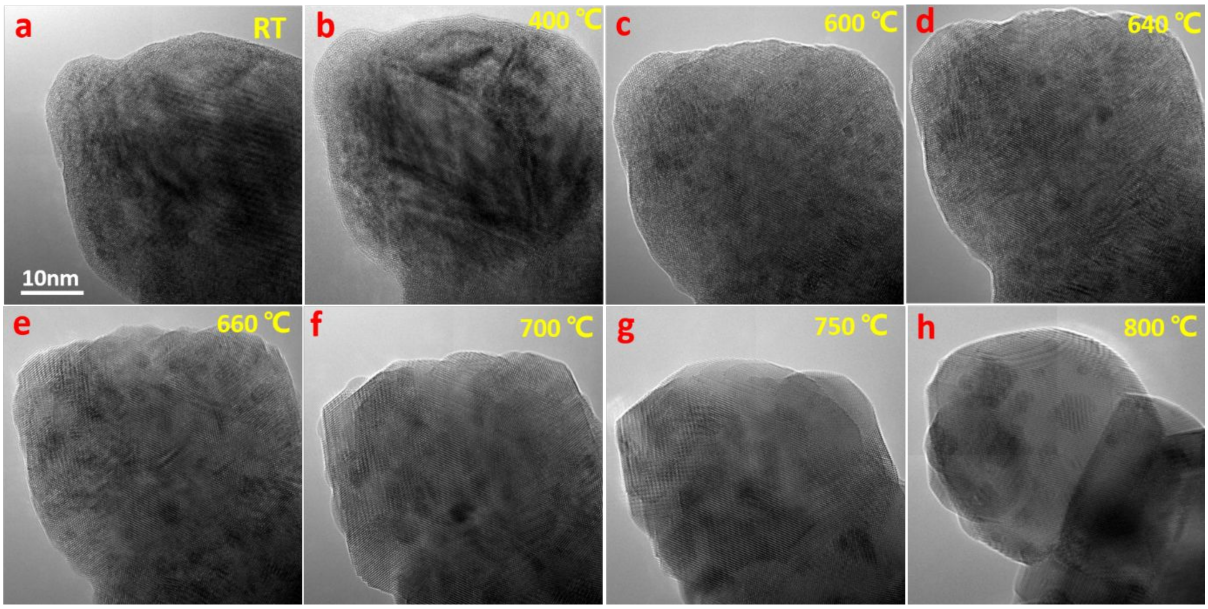


**Figure S9** Series of high-resolution TEM images of $LaNiO_3$ in [001] orientation during the vacuum heating experiment. The representative images show the structure evolution at different temperatures.

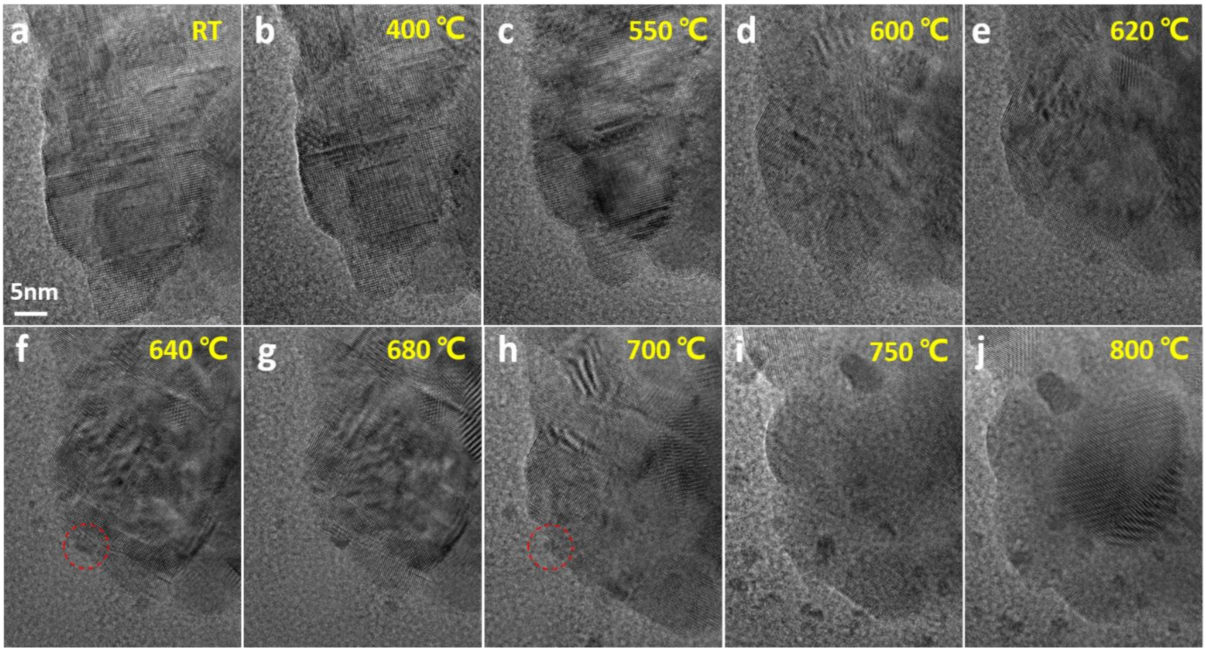


**Figure S10** Series of high-resolution TEM images of $LaNiO_3$ in [241] orientation during the vacuum heating experiment. The representative images show the structure evolution at different temperatures.

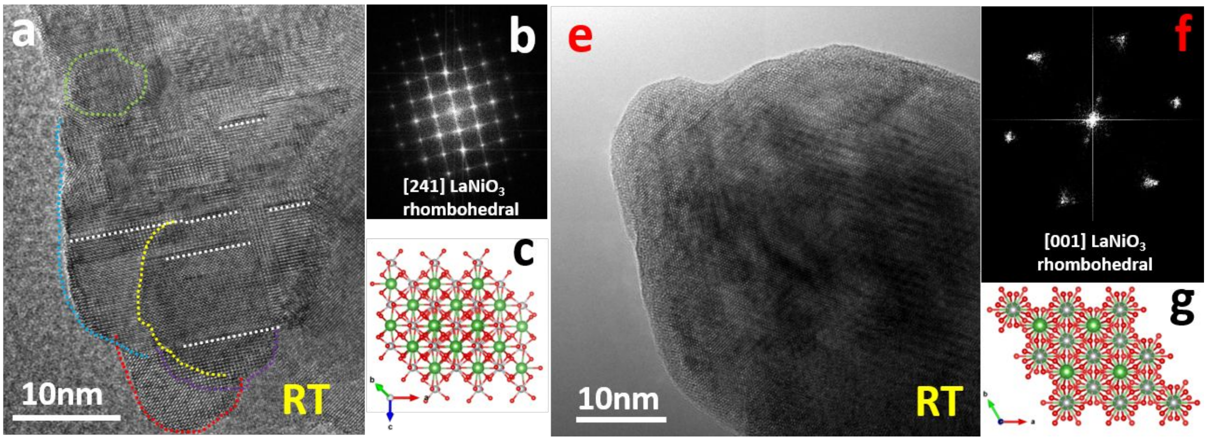


**Figure S11** Initial room-temperature structure of $LaNiO_3$ imaged in two different crystallographic orientations at room temperature: (a) bright-field TEM image in [001] direction. In this orientation, stacking faults (marked by white dashed lines) and grain boundaries (labeled by color dashed lines) were imaged, (b) corresponding FFT in this

orientation, and (c) crystallographic representation, (e) bright-field TEM image in [241] direction, (f) corresponding FFT, and (g) crystallographic representation.

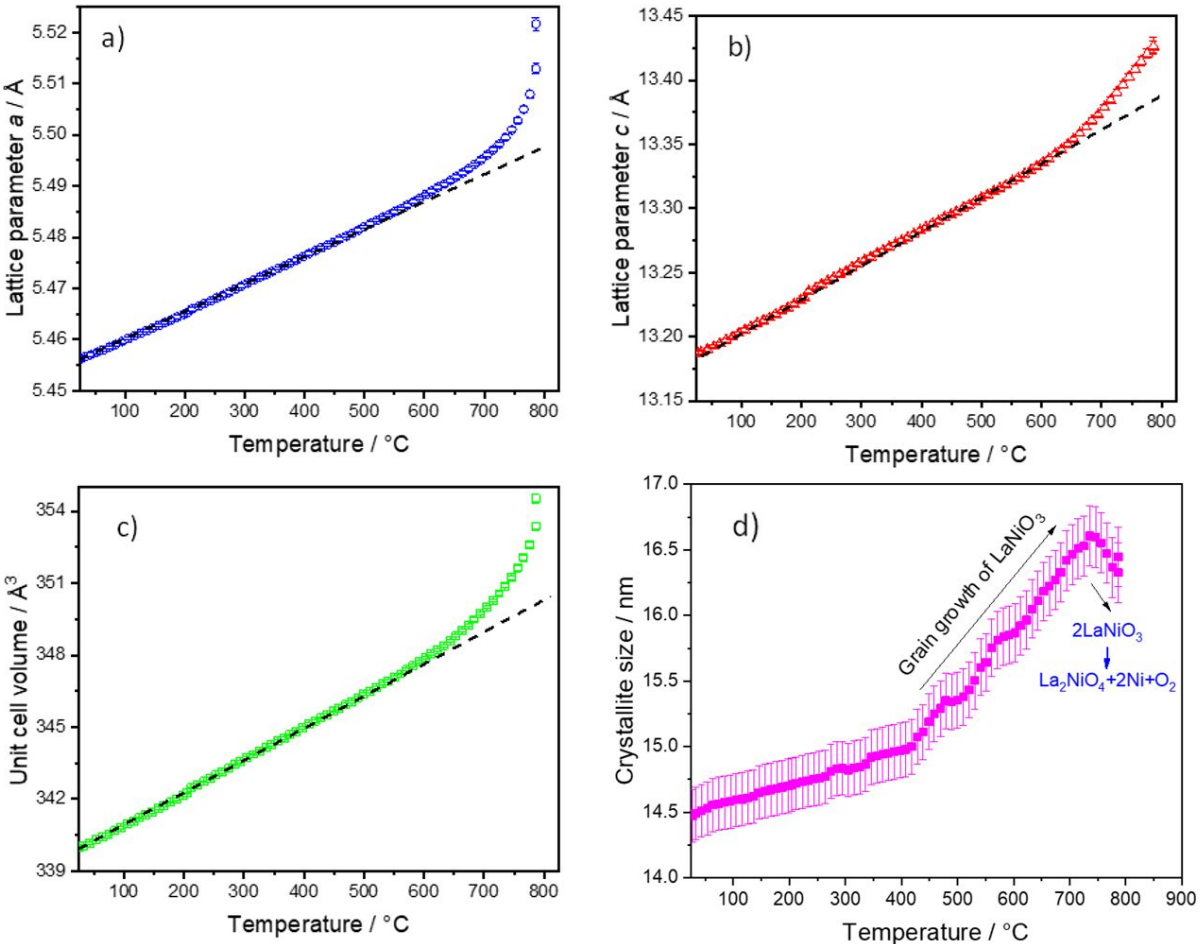


**Figure S12** Evolution of the (a) lattice parameter a, (b) lattice parameter c, (c) unit cell volume, (d) crystal size of $LaNiO_3$ samples as a function of reaction temperature. The dashed black line represents the estimated linear thermal expansion of $LaNiO_3$.

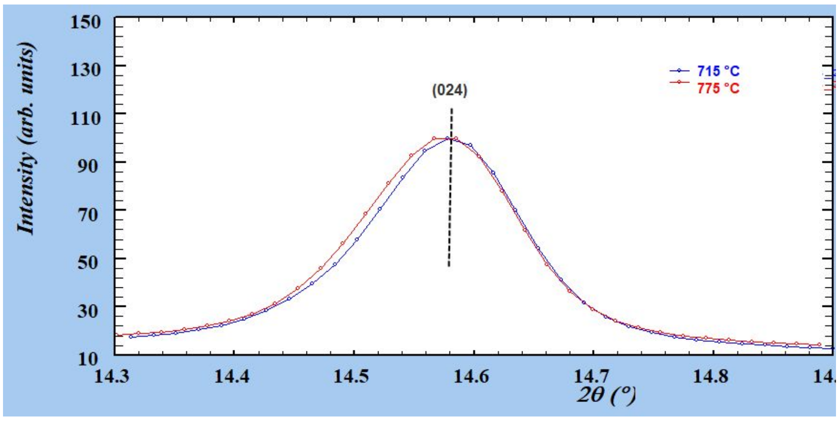


**Figure S13** The XRD reflections correspond to the (024) lattice planes of the $LaNiO_3$ phase at 715°C and 775 °C, respectively. The increase of the FWHM of the reflection with temperature indicates the decrease in the crystallite size of $LaNiO_3$.

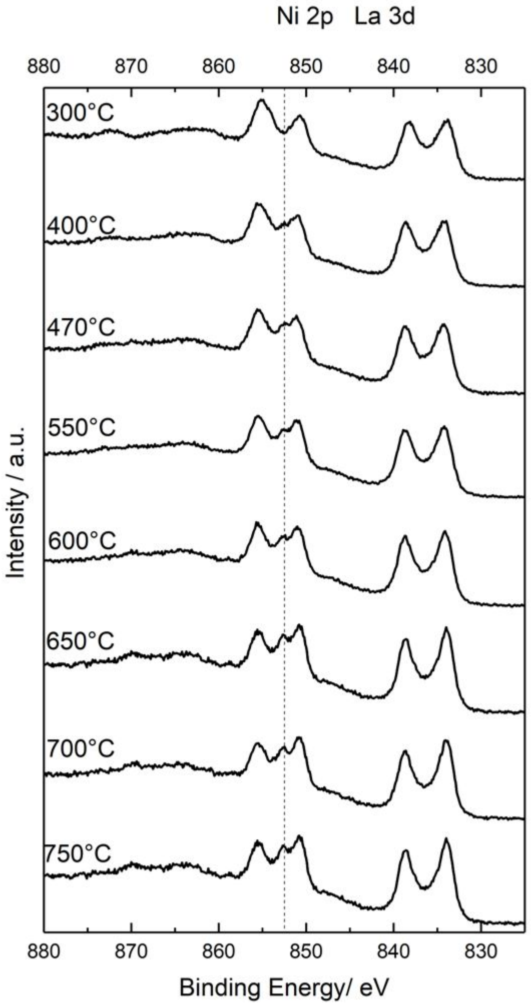


**Figure S14** In situ X-ray photoelectron spectroscopy analysis of the Ni 2p and La 3d region for heating $LaNiO_3$ in 0.2 mbar $H_2$ from room temperature to 750 °C.
